# Breast health awareness campaign and screening pilot in a Kenyan County: Findings and lessons

**DOI:** 10.1002/cnr2.1480

**Published:** 2021-07-08

**Authors:** Valerian Mwenda, Joan‐Paula Bor, Hannah Gitungo, Lydia Kirika, Richard Njoroge, Beatrice Mugi, Daniel Ojuka, Mary Nyangasi

**Affiliations:** ^1^ National Cancer Control Program Ministry of Health Nairobi Kenya; ^2^ Radiology and Diagnostic Imaging Department Kenyatta National Hospital Nairobi Kenya; ^3^ Department of Surgery University of Nairobi Nairobi Kenya

**Keywords:** breast cancer, evaluation, pilot, screening

## Abstract

**Background and Aim:**

Breast cancer is the leading cancer in terms of incidence in Kenya. We conducted a breast cancer awareness and screening pilot to assess feasibility of rolling out a national screening program in Kenya.

**Methods:**

Conducted in Nyeri County during October–November 2019, the pilot had three phases; awareness creation, screening (clinical breast examination and/or imaging) and final evaluation (post‐screening exit interviews and retrospective screening data review). Descriptive statistics on awareness, screening process and outputs were derived.

**Results:**

During the pilot, 1813 CBE, 217 breast ultrasounds and 600 mammograms were performed. Mammography equipment utilization increased from 11% to 83%. Of 49 women with suspicious lesions on mammography, only 22 (44.9%) had been linked to care 4 months after the campaign. Of 532 exit interview respondents; 95% (505/532) were ≥35 years of age; 80% (426/532) had been reached by the awareness campaign. Majority (75% [399/532]) had received information from community health volunteers; 68% through social groups. Majority (79% [420/532]) felt the campaign had changed their behavior on breast health. Although 77% (407/532) had knowledge on self breast examination (SBE); only 13% practiced monthly SBE. More than half (58% [306/532]) had previously undertaken a CBE. Approximately 70% (375/528) were unaware of mammography before the pilot; 86% (459/532) had never previously undertaken a mammogram. Fifty‐five percent (293/532) of respondents had screening waiting times of >120 min.

**Conclusion:**

Community health workers can create breast cancer screening demand sustainably. Adequate personnel and effective follow‐up are crucial before national roll‐out of a breast cancer screening program.

**Abbreviations**
BSE: breast self‐examination
CBE: clinical breast examination
EQ: equipment utilization
KDHS: Kenya Demographic Health Survey
KENCO: Kenya Network of Cancer Organizations
LOS: length of stay
MES: Managed Equipment Services
MOH: Ministry of Health
NCCP: National Cancer Control Program
NCI‐K: National Cancer Institute of Kenya
NCRH: Nyeri County Referral Hospital
NHIF: National Hospital Insurance Fund
UHC: Universal Health Coverage
USD: United States Dollar
WHO: World Health Organization

## 1 BACKGROUND

Breast cancer is the most common cancer among women, affecting over two million women globally and resulting in over 600 000 deaths in 2018.^1^ For effective breast cancer control programs, five key approaches have been described; integration of breast cancer into national cancer control strategic planning by policymakers, development of diagnosis and management guidelines, review of evidence‐based practices by clinicians, identification of priority breast cancer control opportunities by advocates and implementation research training and mentorship.^2^

Breast cancer is the leading cause of cancer morbidity in Kenya, constituting approximately 13% of all cancer cases; and the third leading cause of cancer deaths with approximately 2600 deaths in 2018.^3^ Even in tertiary facilities, about a third of breast cancer cases are diagnosed in stage four, with metastases to bone, brain, lung or liver.^4^ This is associated with high costs of treatment and low overall survival rates. In Kenya, breast cancer occurs earlier in women between ages 35 and 45 years which is 10–15 years earlier than the peak incidence in developed countries.^5^ Knowledge on approaches for early detection of breast cancer is low, especially in the rural areas.^6^

Kenya does not have a mass breast cancer screening program at the population level yet; screening is currently opportunistic and individual‐based. The Kenya National Cancer Screening Guidelines 2018 identify breast cancer as one of the cancers planned for population‐based screening.^7^ The World Health Organization (WHO) recommends conduction of a pilot before launch of a cancer screening program, to guide implementation.^8^, ^9^ In 2016, through the Managed Equipment Service (MES) project, the Ministry of Health availed mammography equipment in all the 47 counties in Kenya. However, there were concerns about the low utilization of the equipment for breast cancer screening and early diagnosis. Therefore, the National Cancer Control Program conducted a breast cancer awareness and screening pilot, to assess the feasibility of utilizing mammography equipment available at county referral facilities to support a national, population‐based breast cancer screening program.

## 2 METHODS

The breast health awareness and screening pilot was a 2 month intervention launched in Nyeri County in October 2019 and ran until November 2019. The pilot had two phases; awareness creation, and linkage to screenings services. The pilot involved community mobilization, training of healthcare workers in clinical breast examination, conduction of mammograms, biopsy taking, as well as monitoring and evaluation.

The campaign was overseen and managed by a partnership between the Nyeri County Government, the National Prevention, Screening and Early Detection of Cancer Technical Working Group under the Ministry of Health's National Cancer Control Program (NCCP), National Cancer Institute of Kenya (NCI‐K) and the Kenya Network of Cancer Organizations (KENCO). Support for the training of healthcare workers was provided by the Surgical Society of Kenya (SSK) and Kenya Association of Radiologists (KAR). The pilot targeted women aged 35 years and above with information on breast health and an invitation to the County Referral Hospital (NCRH) for screening.

### 2.1 Campaign awareness approaches

To reach the target audience, multiple channels were used; including community leaders, community health volunteers (CHVs), mass media (radio and television), social media (Whatsapp and Facebook), advertising materials (leaflets, printed t‐shirts, lesos and posters), health facility information activities and the campaign launch event itself, that brought together key stakeholders.

### 2.2 Pilot evaluation

Evaluation was structured around the two phases of the pilot process; awareness creation and screening process. The outcome evaluation assessed various variables during and after the pilot period. Screening service statistics were compared with a baseline survey conducted in April 2019 based on retrospective review of mammography and breast cancer health records for the period April 1, 2018 to March 31, 2019 at the Nyeri County Referral Hospital.

Specifically, the evaluation assessed increase in mammography equipment utilization, increase in patient throughput, breast cancer detection rate and referral rates. To gauge awareness campaign penetration and effectiveness, exit interviews using a semi‐structured questionnaire were conducted to clients who had undergone mammography during the pilot. The questionnaire had undergone pre‐testing and utilized in a prior breast cancer awareness and practice survey conducted a year earlier. A qualitative assessment of the health system enablers and barriers for breast cancer screening were also performed.

### 2.3 Mammography and equipment utilization

At baseline, equipment utilization was calculated by considering the average number of mammograms per day against the maximum daily mammography capacity of the facility. The calculation was done as shown below:

$$\text{Equipment utilization}\;\left(\mathrm{EQ}\right)=\frac{\text{Average number of mammograms}\;\mathrm{per}\;\mathrm{day}}{\text{Maximum daily mammography}\;\text{capacity of the facility}}\times 100$$

### 2.4 Data management

Descriptive statistics were calculated to describe the changes in awareness and uptake, comparing the baseline with post‐awareness campaign periods. The screening process was described through calculation of detection rates (proportion of women with abnormal findings, out of all women screened) and linkage to care rates (women with evidence of successful linkage to care after positive screening results). Data analysis was conducted using Epi Info™ statistical software, version 7.2 (Centers for Disease Control and Prevention, Atlanta, GA).

## 3 FINDINGS

### 3.1 Screening process and linkage to care

**FIGURE 1:**
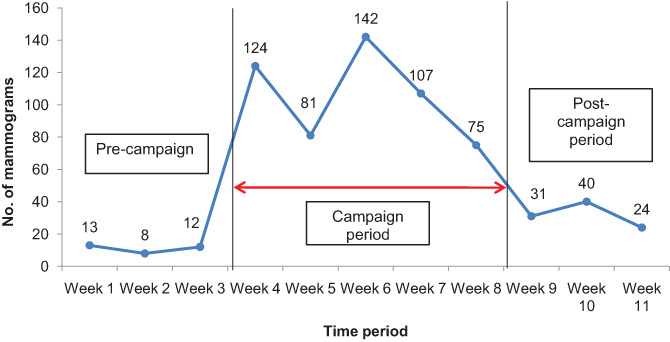
Trends in number of mammograms performed, Nyeri County Kenya; October–November 2019

During the pilot period, 217 ultrasounds were performed, 600 mammograms were undertaken and 1813 women received a clinical breast exam (CBE). There was a 614% increase in the number of clients undergoing mammography in October and November 2019 (600) compared to the same period in 2018 (84), *P* < .0001. The average number of mammograms conducted per day increased from 2 in 2018 to 15 in 2019. The number of mammograms performed decreased sharply after the end of the awareness campaign (Figure 1).

### 3.2 Length of stay during screening visits

**FIGURE 2:**
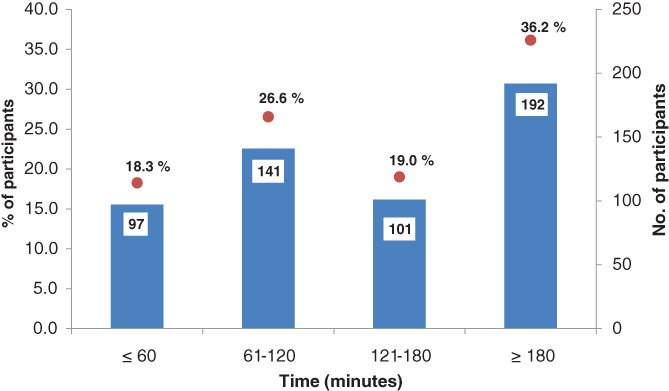
Length of stay at screening mammography service points, Nyeri County, Kenya, October–November 2019

The average length of stay (LOS) for 36% of the clients was more than 180 min (3 h) while 27% and 19% of clients spent 61–120 min and 121–180 min, respectively (Figure 2). Only 18% of the patients indicated that they had spent less than 60 min at the screening health facility. This is compared to a report from healthcare workers during the baseline survey conducted in April 2019, which estimated the LOS at 60 min.

### 3.3 Mammography equipment utilization

Based on interviews at the facility, it was established that a maximum of 18 mammograms could be undertaken per day (three per hour for 6 h in a day). On average, two mammograms were performed per day before the pilot; therefore, equipment utilization was calculated as:

$$\text{Equipment utilization}\;\left(\mathrm{EQ}\right)=\frac{2}{18}\times 100=11.1\%$$

During the pilot, an average of 16 mammograms was performed per day; therefore the equipment utilization was:

$$\mathrm{EQ}=\frac{15}{18}\times 100=83.3\%$$

Therefore, mammography equipment utilization increased from 11% before the pilot to 83% during the pilot period. However, this was not sustained after the pilot, since the average number of mammograms performed per day begun to rapidly decline post‐campaign. In the week following the campaign's end, on average six mammograms were performed per day (EQ = 33.3%).

**FIGURE 3:**
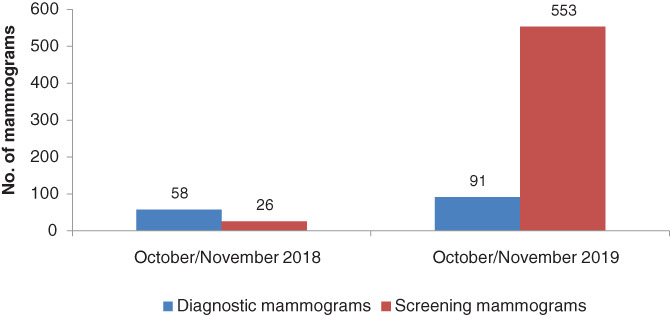
Comparison between screening and diagnostic mammograms performed in Nyeri County Kenya; October/November 2018 versus October/November 2019

While in October and November 2018 diagnostic mammograms were more than screening mammograms, in 2019, screening mammograms were more than diagnostic mammograms (Figure 3).

### 3.4 Breast cancer diagnosis and linkage to care

A total of 49 clients had lesions suspicious for breast cancer on mammography or ultrasound. Four months after the campaign, 22 (44.9%) had undergone biopsies and had been linked to definitive care (either surgery, chemotherapy or both), 5 (10.2%) were awaiting diagnosis with fine needle aspirate (FNA) or biopsy while 22 (44.9%) had not yet gone for biopsy or could not be reached through the phone numbers they provided. A further 25 clients had a provisional diagnosis of fibroadenoma; 9 (36.0%) had undergone further evaluation successfully, 6 (24.0%) were awaiting FNA at the time of pilot evaluation, while 10 (40.0%) had not sought further diagnostic care or were not reachable on phone. Concerns were raised for cases that required surgery since the NCRH was fully booked up to mid‐2020, due to overwhelming demand for surgical treatment at the facility. Four months after the pilot period, out of 600 mammogram reports, 175 (29%) had not been collected by the screened clients.

### 3.5 Post‐screening exit interview/survey

#### 3.5.1 Socio‐demographic characteristics of survey respondents

The exit interview had 532 respondents. Majority of the respondents (76%) were between the ages 30–59 years age (Table 1). Majority (70%) were married and 76% had at least attained primary education and above. Over half (58%) of the respondents were farmers.

**TABLE 1:** Socio‐demographic characteristics of the exit interview respondents, Nyeri County breast cancer awareness and screening pilot, 2019 (*n* = 532)

Variable	Category	Frequency	%
Age	30–39	25	4.7
	40–49	151	28.4
	50–59	228	42.9
	60 and above	128	24.1
Marital status	Single	72	13.5
	Married	372	69.9
	Separated	18	3.4
	Divorced	2	0.4
	Widow	68	12.8
Education level	None	26	4.9
	Primary incomplete	102	19.2
	Primary complete	128	24.1
	Secondary incomplete	58	10.9
	Secondary complete	146	27.4
	Vocational training	17	3.2
	College incomplete	7	1.3
	College complete	48	9.0
Occupation	Farming	308	57.9
	Employed	51	9.6
	Business	124	23.3
	Other	26	4.9
	None	23	4.3

#### 3.5.2 Awareness campaign performance

Eighty percent of the respondents to the client exit survey recalled seeing or hearing of a breast health campaign over the month of October 2019 without any prompting. Approximately 95% of these respondents were in the ‘target group’ (women aged 35 and above). Majority of the respondents (75%) reported that they had received information of the campaign from the CHVs, while 68% mentioned church announcements and Whatsapp groups. Majority of the respondents (72%) reported that the campaign had changed their behavior on breast health.

### 3.6 Early detection, screening knowledge and behavior

#### 3.6.1 Breast self examination

**FIGURE 4:**
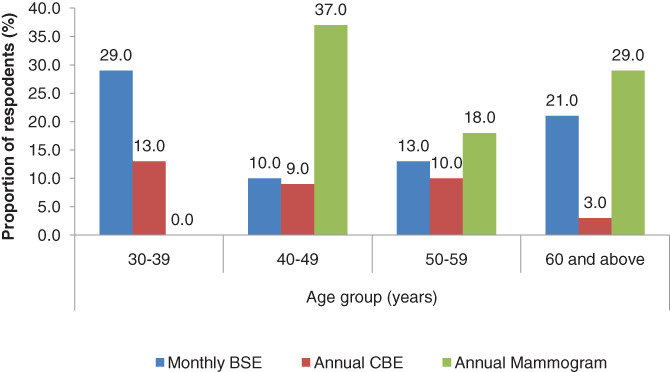
Breast cancer screening awareness and practice, Nyeri County, October–November 2019

Majority of the respondents (77%) had knowledge of breast self examination (BSE). On frequency of performing BSE, majority (75%) of the respondents did not regularly practice BSE; only 13% reported that they practiced monthly BSE (Figure 4). Twenty‐eight percent (7/25) of women of age less than 40 years performed BSE monthly, compared with 14.2% (72/507) of those above 40 years (*P* = .058).

#### 3.6.2 Clinical breast examination

More than half (58%) of the respondents in the exit survey had previously undertaken a clinical breast exam (CBE). Majority (85%) of the respondents reported that they did not undergo CBE mainly because they did not have problems with their breasts; 9% reported that they did not know about CBE while 6% were not comfortable with the exercise. Annual CBE was undertaken by 12.0% (3/25) of those below 40 years and 8.1% (41/507) of those 40 years and above (*P* = .490).

### 3.7 Mammography

Around 71% were not aware or did not know what mammography is before the campaign. Majority of the respondents (87%) had never previously undertaken a mammogram. Majority (78%) of the mammograms undertaken pre‐campaign by the respondents were diagnostic mammograms. Around 73% of the respondents reported that they had not undertaken a mammogram because they did not have a problem with their breast and therefore did not know that they needed one. Other reasons included that a doctor or other health worker had not ordered one (12%) and issues of cost (3%). On frequency of mammograms, all the women under 40 years (100.0%) had a mammogram in the previous complete year, compared to 17.9% (91/507) of those 40 years and above (*P* < .0001).

### 3.8 Payment for mammography‐based screening

At baseline (April 2019) and during the campaign period, it was found that the cost of a mammogram at NCRH was KES 2000 (USD 20). The cost was fully covered for Nyeri County residents registered under the Universal Health Care (UHC) pilot project, taking place at the time. Majority (98%) of respondents who had mammograms during the campaign period did not pay for the service. The rest paid by cash or through the National Hospital Insurance Fund (NHIF).

### 3.9 Screening process experience

Ninety‐nine percent (527/532) indicated that they were satisfied with mammography services at the screening facility and would go back for a subsequent mammogram. Around 87% (463/532) of the respondents indicated they had received respectful care by the person performing their mammogram while 79% (420/532) termed the screening process as comfortable.

### 3.10 Health system readiness evaluation

The health system structures to support breast cancer screening were evaluated based on the WHO building blocks for health systems. The findings are presented in Table 2. Gaps were noted in human resources, health system capacity to support mammography‐based breast cancer screening and an efficient health information system that can track clients through the entire continuum.

**TABLE 2:** Health system readiness evaluation to support mammography based breast cancer screening, Nyeri County, Kenya, 2019

Pillar	Findings
Health information systems	A large number of mammogram reports were uncollected 4 months after the pilot. Data capture was fragmented and findings not fully linked with information captured in the screening registers
Medical products and technologies	The two main supplies that were inadequate at the facility during the campaign period were mammography films and thermoluminescent dosimeters (TLDs) for the radiology staff. The shortages worsened as the number of mammograms performed increased during the pilot
Human resources for health	Before the pilot, the facility had one radiologist and two radiographers competent enough to perform mammograms. During the pilot, one radiologist and two radiographers were added; however, the workload was still more than this team could handle efficiently
Service delivery	Awareness campaign created immediate demand. However, since mammography is available only at the county referral facility, women had to endure significant distances and long‐waiting times to access screening. The approach had to be adapted during the pilot to use CBE as a triaging for the women prioritized for mammography
Health financing	Most of the mammograms performed were free, since Nyeri county was also piloting UHC. However, after the end of the UHC pilot, this has not been sustained
Leadership and governance	The pilot involved ensuring existence of policy frameworks combined with effective oversight, coalition building, regulation, attention to system design and accountability. A close‐out forum with all the implementing agencies provided a model for stakeholders to adopt in the future planning, implementation and review of campaigns. Screening was guided by the National Cancer Screening Guidelines

## 4 DISCUSSION

### 4.1 Summary of findings

During the breast cancer awareness and screening pilot, the average number of mammograms performed increased and equipment utilization peaked; however, this was not sustained after the pilot period ended. Mammography screening for every woman invited for screening was found to overwhelm the screening facility and contributing to lengthy waiting times before undergoing screening and delayed reporting of results; a triage with CBE was deemed more feasible. Limited availability of human resources for mammography screening was a major impediment to optimal equipment utilization and reporting during this pilot. Approximately 45% of women with suspicious lesions for breast cancer and 40% of those with suspected benign conditions were lost to follow‐up before diagnostic work‐up could be done. Community strategy utilizing community health workers was the most effective awareness creation and community mobilization approach; this can also be utilized to track screened women and reduce loss to follow.

### 4.2 Screening process and client experience

A co‐test strategy of CBE and mammography (initial use of CBE and risk stratification before mammography) had to be adopted due to overwhelming demand following the awareness campaign. Majority of the clients had screening turn‐around times exceeding 1 h for the screening mammography procedure. This was longer than what was reported by healthcare personnel before the pilot, despite the fact that more personnel were deployed to meet the expected increase in demand due to the awareness campaign. There was also an additional period of at least 2 weeks for reporting of the mammograms. This could lead to loss to follow‐up and ineffective linkage to further evaluation and/or management. Time factor alone is a key factor in breast cancer screening uptake and adherence to follow‐up.^10^ A CBE‐based national screening program, with linkage to mammography for those with abnormalities or family history of breast cancer has demonstrated effectiveness and practicality in LMIC settings, in terms of target population coverage, linkage to further evaluation and management and down‐staging.^11^, ^12^, ^13^

### 4.3 Breast cancer diagnosis and linkage to care

A loss to follow‐up rate of 40% of those eligible for further diagnostic workup was recorded in this pilot. One of the reasons may be the long‐waiting times for mammography findings to be available; this demonstrates one constraint for mammography‐based screening in resource‐limited settings. Due to the overwhelming number of mammograms performed at the county referral hospital, and limited number of radiologists and radiographers, delayed reporting resulted in poor linkage to care and follow‐up. Trained personnel in mammography were inadequate to support mammography as the primary screening approach at the population level. Also, mammography equipment and radiologists are only available at the county referral facilities; therefore a primarily‐mammography‐based screening approach may be unavailable to majority of women in the population. A CBE‐based screening program in Tajikistan, involving training of various cadres of healthcare providers and integrated into routine care of female clients demonstrated a more efficient process and linkage to further evaluation and management.^14^

### 4.4 Awareness creation approach

Majority of the respondents had been reached by the breast health awareness campaign over the period of intervention. This recall proportion implies that the campaign was effective in its visibility and transmission of the message on breast health awareness to the target group. Community and social media platforms were the most efficient channels for health communication during the campaign. These low‐cost strategies may offer alternatives for cost‐effectiveness and sustainability of cancer control health campaigns, especially in resource‐constrained settings. While mass media approaches in increasing breast health awareness and uptake of screening has been demonstrated in other settings,^15^, ^16^ cost and sustainability in LMIC settings is a major barrier to their wide adoption. Utilization of community structures offers the most sustainable awareness creation and screening invitation approaches in LMIC settings or even high‐income settings with health disparities.^17^, ^18^

### 4.5 Early detection, screening knowledge and behavior

Majority of the respondents had knowledge on the two approaches for breast awareness; self breast examination and clinical breast examination. However, fewer women carried out the two examinations at the recommended frequency and regularity. Most of the respondents assumed SBE and CBE are performed when one has medical complaints about the breast. More than half of the respondents had previously undertaken a clinical breast exam, which is higher than the most recent national average of 14%.^19^

Knowledge on mammography was low among the respondents. Therefore, underutilization of mammography for screening may be driven by low public awareness of mammography and its availability at county referral hospitals. Majority of the respondents had never previously undertaken a mammogram and this awareness and screening campaign was their first ever screening mammogram. Majority of the mammograms undertaken pre‐campaign by the respondents were diagnostic mammograms. Utilizing the healthcare system structures like integration of screening invitation as women seek other services may be an effective approach for countries launching breast cancer screening programs.^20^, ^21^, ^22^, ^23^

### 4.6 Health system opportunities and barriers to breast cancer screening

During the pilot, the pilot county was under a Universal Health Coverage pilot phase; therefore all screened women, who were residents of the County, did not pay out‐pocket for mammography. However, since the end of the UHC pilot phase, a financing mechanism for national roll‐out is not yet finalized; therefore at the moment, majority of women in all the Kenyan counties would require to pay for the mammography. This may not be sustainable to offer screening at population level since cost of screening tests is a major cause of low uptake. Even with coverage of the cost of mammography, the screening facility experienced stock‐out of radiological films and other essential supplies. Only the county referral facility has mammography equipment. Human resources at the imaging department were strained during the pilot due to heavy work‐load. Breast cancer screening must be situated within the context of the national healthcare system, recognizing the realities and barriers, for sustainability and effectiveness.^24^

### 4.7 Strengths and limitations

A particular strength of the approach employed during this pilot was the combination of screening process data and feedback from screened clients. This would further inform the planned national roll‐out of breast cancer screening program. However, the pilot study had some limitations. First, the pilot intervention period was rather short. Therefore, it was not possible to evaluate the medium term effects of the awareness creation. Second, a component of formative research would have given more insights into the target population attributes that would impact breast cancer screening uptake.

## 5 CONCLUSION

A community awareness and provision of information on breast cancer screening can create demand; however, the healthcare system needs to be well prepared to offer the screening and linkage to care to all women seeking screening services. All the pillars of healthcare systems strengthening must be improved to support an effective breast cancer screening program. A CBE‐based screening, with linkage to imaging may be the most feasible approach as breast cancer screening is introduced at the population level in Kenya even as we focus on increasing staff training and availability to provide screening services.

## CONFLICT OF INTEREST

The authors declare that they have no conflicts of interests.

## AUTHOR CONTRIBUTIONS

All authors had full access to the data in the study and take responsibility for the integrity of the data and the accuracy of the data analysis. *Conceptualization*, V.M., J.‐P.B., B.M., M.N.; *Methodology*, V.M., R.N., M.N.; *Investigation*, L.K., R.N., B.M., D.O., M.N.; *Formal Analysis*, V.M.; *Writing—Original Draft*, V.M.; *Writing—Review & Editing*, V.M., D.O., M.N.; *Supervision*, H.G., M.N.; *Data Curation*, V.M.; *Validation*, V.M.; *Project Administration*, J.‐P.B., H.G.; L.K.

## ETHICAL STATEMENT

The pilot project was part of the planned roll‐out of breast cancer screening in Kenya, as recommended by the World Health Organization. Therefore, the pilot was authorized by the Ministry of Health as a routine surveillance and programmatic undertaking to inform phased implementation from 2021, and was not subjected to an institutional review board process. Screening information was collected following routine cancer surveillance structures in Kenya. However, verbal consent was sought from the exit interview respondents before conduction of the interviews, after explanation of the study rationale.

## Data Availability

The data that support the findings of this study are available from the corresponding author upon reasonable request.
